# Public and patient involvement in needs assessment and social innovation: a people-centred approach to care and research for congenital disorders of glycosylation

**DOI:** 10.1186/s12913-017-2625-1

**Published:** 2017-09-26

**Authors:** Cláudia de Freitas, Vanessa dos Reis, Susana Silva, Paula A. Videira, Eva Morava, Jaak Jaeken

**Affiliations:** 1 EPIUnit - Instituto de Saúde Pública, Universidade do Porto, Porto, Portugal; 2 Institutional address 1: Rua das Taipas 135, 4050-600, Porto, Portugal; 3 Centre for Research and Studies in Sociology - University Institute of Lisbon, Porto, Portugal; 4 Institutional address 2: Avenida das Forças Armadas, 1649-026, Lisbon, Portugal; 5 Founder of the Portuguese Association for CDG (APCDG), Porto, Portugal; 6 Institutional address: Rua Manuel da Fonseca 46, 2820-389, Almada, Portugal; 7 Faculdade de Ciências e Tecnologia, Universidade Nova de Lisboa, Lisbon, Portugal; 8 Institutional address: Glycoimmunology group Lab 3.19 - Departamento Ciências da Vida (Ed Departamental), Faculdade de Ciências e Tecnologia, 2829-516 Caparica, Portugal; 9 School of Medicine, Tulane University, New Orleans, USA; 10 Institutional address: Hayward Genetics Center SL#31, Tulane University Medical School, 1430 Tulane Ave, New Orleans, LA 70112 USA; 11 Department of Pediatrics, Center for Metabolic Disease, University Hospital Gasthuisberg, Leuven, Belgium; 12 Institutional Address: Herestraat 49, 3000, Leuven, Belgium

**Keywords:** Public and patient involvement, People-centred care, Patient-oriented research, Needs assessment, Social innovations, Rare diseases, Congenital disorders of glycosylation, ELSI

## Abstract

**Background:**

Public and patient involvement in the design of people-centred care and research is vital for communities whose needs are underserved, as are people with rare diseases. Innovations devised collectively by patients, caregivers, professionals and other members of the public can foster transformative change toward more responsive services and research. However, attempts to involve lay and professional stakeholders in devising community-framed strategies to address the unmet needs of rare diseases are lacking. In this study, we engaged with the community of Congenital Disorders of Glycosylation (CDG) to assess its needs and elicit social innovations to promote people-centred care and research.

**Methods:**

Drawing on a qualitative study, we conducted three think tanks in France with a total of 48 participants, including patients/family members (*n* = 18), health care professionals (*n* = 7), researchers (n = 7) and people combining several of these roles (*n* = 16). Participants came from 20 countries across five continents. They were selected from the registry of the Second World Conference on CDG through heterogeneity and simple random sampling. Inductive and deductive approaches were employed to conduct interpretational analysis using open, axial and selective coding, and the constant-comparison method to facilitate the emergence of categories and core themes.

**Results:**

The CDG community has unmet needs for information, quality health care, psychosocial support and representation in decision-making concerned with care and research. According to participants, these needs can be addressed through a range of social innovations, including peer-support communities, web-based information resources and a CDG expertise platform.

**Conclusion:**

This is one of the few studies to engage lay and professional experts in needs assessment and innovation for CDG at a global level. Implementing the innovations proposed by the CDG community is likely to have ethical, legal and social implications associated with the potential donation of patients’ clinical and biological material that need to be assessed and regulated with involvement from all stakeholders. To promote people-centred care for the CDG community, and increase its participation in the governance of care and research, it is necessary to create participatory spaces in which the views of people affected by CDG can be fully expressed.

## Background

Public and patient involvement is a fundamental element in implementing patient-oriented research and improving the design and provision of people-centred care [1–3]. By enabling lay and expert knowledge to come to the fore, public and patient involvement can help promote needs-driven research, as well as care practices centred on people’s needs, values and preferences [4, 5]. This is particularly relevant for people with rare diseases whose needs are often underserved [6–9]. Yet, only a few studies have focused on identifying patient-framed solutions for the unmet needs of rare diseases [6, 10], and attempts to involve both lay and professional experts in dialogue about innovations to address them appear to be lacking. Disregarding public and patient perspectives risks undermining the societal relevance and acceptability of research [11], as well as health care systems’ responsiveness to people’s right to quality care [12]. In this study, we engaged with the community of Congenital Disorders of Glycosylation (CDG) to assess its needs and elicit its views on how to achieve people-centred care and research.

CDG are a group of rare inherited metabolic diseases caused by defects in glycosylation [13]. Glycosylation is present in all tissues and organs. Consequently, errors in glycosylation can have a large spectrum of consequences (e.g. affecting the muscular, cardiovascular, immune and central nervous systems) [14] and clinical manifestations (e.g. cardiac disease, severe developmental delay) [15–17]. Signs and symptoms typically present from infancy and vary across CDG types [18]. The most common type of CDG – PMM2-CDG – has an estimated incidence of 1:20,000 live births, affecting males and females in a similar way [19]. Mortality in the early years is approximately 20% [18]. Affected patients can survive into adulthood, but dependence on care services and caregivers is high throughout the lifespan [18]. Although CDG is one of the fastest growing groups of monogenetic diseases [20], little investment has been made in treatment development and most CDG types lack an effective therapy [21]. To reduce the burden of CDG, it is necessary to involve patients, caregivers, professionals and other members of the public in setting priorities for research and developing care centred on patients’ needs.

Eliciting community-framed solutions is expected to advance tailor-made innovations and effect transformative change [22]. When these innovations are geared towards solving complex social problems such as overcoming services’ limited responsiveness they can be termed social innovations [23]. Social innovations have the potential to harness latent or unrealised value [24] by fostering collaboration between previously disconnected groups and uncovering new understandings that challenge preconceived ideas [25]. In other words, they can join stakeholders from various backgrounds, and promote public and patient involvement in health decision-making.

Drawing on a qualitative study that deployed think tanks with lay and professional stakeholders, this paper delves on the experiences of the CDG community to identify its unmet needs and explore innovations that may help address them. In doing so, it aims to facilitate the emergence of alternative forms of knowledge, expand the evidence base for the needs of people affected by rare diseases, and elicit tailor-made innovations to foster change toward quality care and research.

## Methods

A qualitative study was undertaken deploying three think tanks – a form of group interviews particularly suitable to inquire and elicit debate on previously defined topics among large and heterogeneous stakeholder groups [26, 27]. A semi-structured interview guide was used in all think tanks. It addressed two topic questions: 1) what are the challenges experienced by people living with or caring for patients with CDG? And, 2) what strategies can be used to overcome those challenges? Research ethics approval was obtained following project approval by the Foundation for Science and Technology (Portuguese Ministry of Science, Technology and Higher Education). The study followed the Code of Ethics of the International Sociological Association. All participants provided prior written informed consent to participate in the study and to audio record the think tanks. Think tanks were held in August 2015, in Lyon, France, during the Second World Conference on CDG in a venue specifically assigned for that purpose.

### Data collection

Participants’ selection involved the sequential employment of probability and non-probability sampling techniques [28] using parameters collected from the registry of the Second World Conference on CDG. Heterogeneity sampling was used to ensure maximum variation regarding participants’ experiences and perspectives based on two criteria: type of connection to CDG (i.e. patients, family members, researchers, health care professionals and people combining several roles) and country of residence. Eighty invitations were sent out by email aiming to get a minimum of fifteen participants per think tank. Participants’ sampling and invitation were done by the conference organisation committee. Forty-nine people responded. They were all contacted by the first author through email to confirm participation and complete a sociodemographic survey.

Only one adult patient responded to the invitation, perhaps because most people affected by CDG experience considerable cognitive and physical impairment, which limit their ability to engage in speaking-based activities. To protect the confidentiality of the patient’s statements, the categories “patient” and “family member” were grouped together. Participants were further divided into “researcher”, “health care professional” and “multiple roles”. Subsequently, participants from each group were randomly assigned to one of the think tanks to guarantee a similar distribution per category (see Table 1). One person from the original sample did not show up and another one was re-assigned to an ensuing think tank due to a flight delay.

**Table 1:** Participants’ sociodemographic characteristics

Characteristics	TOTAL *N* = 48	Think Tank 1 (*n* = 16)	Think Tank 2 (*n* = 15)	Think Tank 3 (*n* = 17)
Type of participants				
Patient/Family Member^a^	18	6	5	7
Researcher (clinical or non-clinical)	7	5	1	1
Health Care Professional	7	1	4	2
Multiple Roles^b^	16	4	5	7
Country of Residence (continent)^c^				
Europe	31	10	10	11
North America	11	3	3	5
Other^d^	6	3	2	1
Gender				
Female	33	11	10	12
Male	15	5	5	5
Education				
PhD	13	6	4	3
MA/MSc	13	3	2	8
Bachelor/High School	22	7	9	6
Experience CDG (Years)				
<5	22	7	6	9
5–9	12	4	4	4
≥ 10	14	5	5	4
Involvement in patient organisation				
Yes	24	6	7	11
No	24	10	8	6

^a^It includes 1 patient and 17 family members

^b^It includes participants with more than one role: family member and non-clinical researcher (n = 1); family member and health care professional (*n* = 4); clinical researcher and health care professional (*n* = 8); family member, clinical researcher and health care professional (*n* = 3)

^c^It includes the following countries: Australia, Belgium, Canada, Chile, Czech Republic, Denmark, Finland, France, Germany, Ireland, Israel, Italy, Netherlands, Slovak Republic, Spain, Sweden, Turkey, UK, USA, and United Arab Emirates

^d^It includes participants from Asia (*n* = 2), Australia (n = 2) and South America (n = 2)

All think tanks were conducted in English. They lasted ninety minutes on average and were led by one moderator (CF) together with two observers (PV, JJ) who summarised the main discussion topics at the end of each think tank. Discussion was elicited based on a brief presentation reporting the findings of a study about CDG civic mobilisation in Portugal. Transitioning from one question to the following was done when participants showed to have nothing else to add. Participants were offered the opportunity to comment on think tank summaries and to make final observations. The summaries were also used to assess whether new themes emerged in subsequent think tanks. After the third think tank, no original material was added. This determined theoretical saturation [29], leading to the discontinuation of data collection.

### Participants

Forty-eight participants residing in twenty countries participated in the think tanks (Table 1). The majority were female (33/48) and had a post-graduate degree (26/48). The sample included eighteen patients/family members, seven researchers, seven health care professionals and sixteen people combining at least two of these roles. Most participants had experience with CDG for less than ten years (34/48) and half were involved in patient organisations.

### Data analysis

Audio files from the think tanks were transcribed verbatim by freelance transcribers and checked for accuracy. The data were analysed with the assistance of NVivo 11 employing inductive and deductive approaches to interpretational analysis. Open, axial and selective coding, and the constant-comparison method [29] facilitated the emergence of inductive categories and core themes. First, the data were broken down into tentative categories (open coding), which were subsequently put together into themes by identifying the connections between them (axial coding). Data from each think tank were then examined to select core themes (selective coding) and iteratively compared with data from other think tanks (constant comparison). This led to the identification of two core themes: participants’ needs (including “information needs”, “health care needs”, “psychosocial needs” and “representation needs”); and, strategies proposed to address them (including “strategies developed by patient and advocacy groups”, “strategies to deal with health and social care needs” and “online strategies to disseminate information and bolster research”). During selective coding, inductive themes were loaded with theoretical sensitivity in consultation with the existing literature [29], i.e. theoretical codes [30] were developed by “infusing” empirical themes with theory. Themes linked to patients’ needs emerged close to the formulations used in the literature requesting no adaptations. Themes associated with “strategies to address challenges” were deductively reformulated as “social innovations” given their resemblance with the concept [24, 25].

Strategies used to guarantee the rigour and quality of research included triangulation of data sources, which were collected in three different moments in time from people with various origins, roles and stakes in the field of CDG, and analyst triangulation, with CF leading the analysis and SS collaborating in the development of the coding framework and in the phase of selective coding. Quotes from the think tanks are presented using aggregate attributes in the case of patients and family members to protect the confidentiality of all participants’ statements.

## Results

The unmet needs experienced by participants and the social innovations proposed to overcome them are illustrated by direct quotes drawn from the think tanks presented in the text and supplemented by Table 2 and Table 3, respectively.

**Table 2:** Unmet health and informational needs identified in think tank discussions – supplementary quotes

1 Information unmet needs
1.1 Limited clinical information
[1.1a]: Multiple Roles 1: “They [parents] don’t know where to get the information and, being so rare, a lot of very good clinicians don’t have that information.”
1.2 Limited information about experimental treatments
[1.2a] Patient/Family Member 5: “There’s a lot of information in the Internet about CDG but we cannot say whether it’s right or wrong. We already talked about Mannose [experimental treatment]. One says that’s the trick and others say: ‘Oh no, that’s not working’.”
1.3 Language barriers
[1.3a] Patient/Family Member 16: “All the info we got about CDG, we found on our own. (…) We found a Facebook CDG community group where we got answers. (…) We would like to disseminate this information for our Czech families. But (…) we don’t have so much time and energy, and we need to translate everything to Czech.”
2 Health care unmet needs
2.1 Problems with diagnosis
[2.1a] Professional 7: “In South America, at least in Chile, (…) if I’m suspecting a patient to have a CDG, I’m not able to request the testing and this is a big issue. I know that the testing is available in Argentina and Brazil, but we’re struggling to send the biological samples across the border (…) because we have more rules for biological samples.”
2.2 Professionals’ limited awareness of CDG
[2.2a] Patient/Family Member 1: “When I have to go to the emergency of a local hospital, they don’t know anything about CDG. (…) And when I got too many side effects, they just said: ‘Everything can be due CDG’. (…) So how can you create more awareness among doctors?” [2.2b] Multiple roles 9: “I feel that in our country [Italy] knowledge about CDG is now rather consolidated among paediatricians and metabolic doctors but it is not a sufficiently known among physicians that have adult patients in their care and also not, for example, among neurologists.”
2.3 Complexities of CDG
[2.3a] Multiple Roles 8: **“**One difficulty for the professionals is that CDG is a rapid and ever-growing group of disorders. So, even for professionals who are really interested in CDG, it’s a real task to be on top of the new diseases. (…) We are all limited for the time and efforts that we can put into the subject. We need to develop information packages that we can resort to.”
2.4 Inexistence of treatment
[2.4a] Multiple roles 1: “Researchers are not as interested [in rare diseases] because it’s hard to bring money in and pharmaceutic companies are not interested because it’s a very small market.”
2.5 Unsatisfying care delivery
[2.5a] Multiple Roles 10: “We have been in three different cities in the United States taking our daughter for care and one challenge that we definitely encounter is that not all doctors want to reach out to someone who may be a medical expert in a rare disease.”
3 Psychosocial needs
3.1 Distress upon diagnosis
[3.1a] Patient/Family Member 14: “We felt really alone [after receiving the diagnosis], because we could not understand what CDG was, what had happened: ‘And now? We go home with our child and what? What future will he have?”
3.2 Diminished quality of life
[3.2a] Patient/Family Member 18: “In the UK we’re really lucky because my son has been given disability living allowance and also [my husband was given] a carer’s allowance. So as long as he [husband] looks after him for over 40 h a week, he gets paid a certain amount of money by the government and also he is allowed to work to up to 16 h a week.” [3.2b] Multiple Roles 11: “There are lot of things [welfare benefits] that are really excellent in Germany. I believe in Sweden it should be as well, even better probably. (…) That might be a good argument to tell the insurance companies: ‘They have more resources so why can’t you give us that?”
4 Representation needs
[4a] Professional 7: “People in Latin America don’t have the culture of associating (…). Also, their model of clinical relationship is a little different from the European or North American. My patients, I always ask if they want to contact other families and usually they don’t want to.”

**Table 3:** Social innovations identified in think tank discussions – supplementary quotes

1 Civil society innovations
1.1 Providing information and support to families [1.1a] Multiple Roles 6: “My doctors didn’t know what was going on and I reached out to one of the families in New Zealand. She [mother] told me she could take our data to her hospital and show it to her physician, and they took my daughter into account (…) and we just immediately got feedback. (…) So getting those resources out to parents would be very helpful.”
1.2 Advocating for social and financial support [1.2a] Patient/Family Member 13: “We’re in such small numbers in each country. I was wondering: ‘Would it make sense to create some sort of CDG Europe?’ (…) Then maybe it would be easier to approach insurance companies. And it actually would be easier to access funding (…)”.
1.3 Promoting research [1.3b] Multiple Roles 16: “It is [important] for physicians across the globe to begin to really collaborate and learn from each other in a very different way that we’ve traditionally done. (…) So I think that’s something else parents and family organizations can push towards. (…) We want lots of places (…) all around the world talking to each other, so that we’re sharing the new things that are getting learned in a faster, easier way.”
2 Care-related innovations
2.1 Increasing professionals’ awareness [2.1a] Multiple Roles 14: **“**If physicians are going to take their board examinations, they will learn about certain diseases, and people learn quickly if they know that they’re likely to be asked about CDG. (…) But another thing (…) is to give conference talks, to publish articles about it.”
2.2 Improving care approaches [2.2a] Professional 3: “I think you have to identify a few important problems that are common within the group of CDG patients and then try to find information that is already there, just a retrospective analysis. And then, maybe try to put forward a few strategies and test them in the population so you can make progress in this disease group.” [2.2b] Multiple Roles 8: “The next thing that should happen is that we exchange what we think about it with doctors in other countries who have treated patients with the same problem. (…) And (…) we can identify what has been done and what was the outcome. This will enable us to get better treatment strategies.”
2.3 Families’ involvement in treatment [2.3a] Patient/Family Member 18: “Sometimes we [parents] just have to think: ´I’m going do it and get over the embarrassment. (…) If I don’t understand, I’m going to keep repeating my question until I get an answer.”
3 Digital innovations
3.1 Developing a CDG expertise online platform [3.1a] Patient/Family Member 11: “Structured online platforms are really the way ahead. Controlled information could be disseminated, questions could be asked but, yes, it does need to be an international platform.” [3.1b] Multiple Roles 8: “It’s important that we have different country representatives because resources and health structures are very different in different countries.” [3.1c] Professional 3: “The information exchanged in a platform like that has to be controlled medically (…) it can’t just be an open platform of communication where information is exchanged. That can sometimes be even dangerous to patients.”

### Needs confronting people affected by CDG

Participants stated that the complexity of and limited knowledge about CDG impose several challenges on people affected by the illness, including insufficient access to information, quality health care and psychosocial support, and limited representation of CDG patients and caregivers in research and civic mobilisation.

### Information needs

Accessing reliable information is a key concern for many CDG families. According to professionals, lack of access to physicians who are knowledgeable about CDG limits families’ ability to obtain accurate information (1.1a). Difficulties in acquiring information, particularly about experimental treatments (1.2a), cause considerable doubt and anxiety to parents:

*“But how do we get hold of the right physicians? How do we get the right information?”* (Patient/Family Member 8).

Some participants overcome informational needs by resorting to social media platforms created by CDG families internationally. However, extending information to all members of the CDG community can be complicated by language barriers (1.3a) and the perils of relying on unverified information, particularly where it concerns the unknown effects of experimental treatments:

*“When patients start exchanging information (…) [experimental] treatments come to play. There’s a lack of robust information about their effects and it can even become dangerous, either medically or financially.” (Professional 3).*

### Health care needs

Unmet needs for health care are associated with the rarity of CDG and the complexity of its clinical presentation. They are especially evident with respect to diagnosis. In the countries and regions where genetic testing is unavailable (2.1a), CDG has extremely low prevalence rates. This suggests that CDG may be under-diagnosed globally. Several participants also stated that they received misdiagnoses or diagnoses with long delays. Failure to obtain a correct diagnosis within a reasonable time frame can have a significant negative impact on patients’ physical and psychological outcomes.

*“It took us 13 years before we got the diagnosis and they [daughters] were both diagnosed 2 years ago. How we got the diagnosis finally was actually a result of co-operation between patient organizations.”* (Patient/Family Member 9).

*“She [daughter] was considered probably to just have a cerebella problem. She was actually tested twice (…) and the results came back negative. So, it wasn’t until she was 14, and she actually had quite a deterioration, that she was tested again.”* (Multiple Roles 3).

Many health care professionals lack knowledge about the existence of CDG (2.2a–b), which often leads to delays in referrals for adequate genetic testing and to under-diagnosing:

*“For a lot of physicians, CDG seems to be quite low on the testing. One, because they don’t know about it. And two, they think: ‘well, maybe it’s not worth it to test because there’s no known treatment’.”* (Multiple Roles 16).

Specialist physicians also express difficulties keeping up with the complexities of the disease. CDG is a multi-system condition that affects many bodily functions and has a variable clinical presentation. This challenges the development of a unified standard approach to deal with the variety of symptoms that may arise during a patient’s life course (2.3a). These difficulties are further intensified by the: existence of ultra-rare CDG types on which there is limited clinical knowledge; unavailability of genetic and clinical expertise in some countries and regions, and restrictive cross-border testing regulations (2.1a); and, resistance by some professionals to contact other medical experts (2.5a).

Research on CDG has not been a priority funding area for the pharmaceutical industry. This limits the amount of investigation done on CDG, as well as clinical trials, which undermines treatment discovery (2.4a). Inability to rely on a treatment increases families’ dependence on health care provision, rendering experiences with inappropriate care delivery especially worrisome and exasperating:

*“We were discharged by the neurologist (…). And I’m like: ‘What? My kid has a condition and one of the main symptoms is a neurological problem and you’re discharging me? (…).’ The lack of knowledge (…) can be threatening.”* (Patient/Family Member 13).

*“We wanted to know [our son’s diagnosis] because we had another son who passed away at 2 months old without known causes. (…) Not getting the information at the right time is very frustrating because (…) within a couple of years one can be dead. (…) From our first experience, unfortunately, we became a little bit less trusting of any doctor.”* (Patient/Family Member 3).

Difficulties in obtaining care adequate to their needs causes concern and frustration to families. Such adversities led some participants to reduce their trust on health care professionals, while others reported going to great lengths to overcome local limitations in expertise by mobilising transnational health care resources:

*“Unfortunately, the country we live in and the country we are from have very limited knowledge and very limited professionals, so we have to do everything on our own. (…) We reached out to Professor Jaak Jaeken [Belgium], to Doctor Patterson [United States]. We had to go to a lab to draw blood, arrange with FedEx and get a doctor’s signature to be able to ship it abroad.”* (Patient/Family Member 2).

### Psychosocial needs

Obtaining a CDG diagnosis was highly distressful for many families given the uncertainty about the child’s prognosis and the family’s future (3.1a). Having a child with CDG often means that one parent has to quit her/his professional career to become a full-time caregiver.

*“I take care of my daughter and they [doctors, teachers] just don't understand (…) that they're clocking in at 8A.M. and they're off at 5P.M. and we are working 24h shifts at home. We don't get breaks at weekends. We work 7 days a week.”* (Multiple roles 10).

Taking a leave from work or quitting altogether impacts families’ incomes on different degrees depending on specific national regulations and existing social supports for long-term medical disability (3.2a–b). Moreover, it impacts families’ quality of life, which can be substantially reduced when emotional, social and financial support are insufficient or lacking:

“*No one can understand what it means for one of the parents to stop his professional life. And apart from that, what is your life? Because our children don’t spend only hours at the hospital, but also with therapies. (…) It’s important for us parents to make society understand that it’s (…) about the quality of life of our family.”* (Patient/Family Member 14).

### Representation needs

Some participants expressed concern that only a limited number of patients and families are actively involved in advocacy activities:

*“I think we have a misrepresentation here [world conference] of parents of CDG patients. (…) It’s great to have all these family members here that are getting involved and participating and sharing information, but that’s a minority.”* (Professional 3).

Patient and public involvement is limited by cultural, financial and geographical barriers (4a) that diminish people’s ability and willingness to engage in peer support, research (e.g. setting priorities) and advocacy – something that participants found was imperative to change:

*“We need to get better resources and better means of getting families that are sitting on the countryside, somewhere in the UK, with very limited financial support to have a voice in the CDG community as well.”* (Multiple Roles 8).

### Social innovations to overcome the unmet needs of people affected by CDG

Participants suggested several innovative strategies to address the needs expressed by the CDG community. Some are concerned with initiatives emerging from and seeking to overcome the needs of patients and families (“civil society innovations”). Others are more directly related to dealing with challenges at the health and social care level (“care-related innovations”). And yet, others entail the use of digital technologies to address the community’s multi-level needs (“digital innovations”) [24].

### Civil society innovations

Participants’ proposals for civil society innovations aim to mitigate the negative effects of unmet needs for information, psychosocial support and representation. Face-to-face and online peer support were considered the most effective ways to assist families in obtaining the information and psychosocial support needed (1.1a). Overcoming representation needs meant reaching out to and involving a more heterogeneous group of families in, and within, different countries. Mobilising CDG families to take part in advocacy groups can help increase their lobbying power with the government and care sponsors (1.2a):

*“I think you have strength in numbers and it might be useful even to think of whether you call it Global CDG. (…) I think when you have a group like that you could also advocate with your government representatives and that’s a very powerful voice.”* (Multiple roles 14).

Engaging in advocacy can also help families connect with physicians and clinical researchers, and increase their information about and participation in international studies (1.3b) concerned with advancing research on CDG.

*“From a doctor’s perspective, (…) if you have one patient, it’s difficult to allocate a lot of interest, time and motivation. (…) So, from a patient’s perspective, you have to identify these people who have more than one patient and then you have to make sure that other patients know as well that they’re interested, motivated, knowledgeable.”* (Professional 3).

### Care-related innovations

Adapting health care provision to the needs of patients and families requires social innovations centred around three goals: increasing professionals’ awareness of CDG, improving care for CDG, and involving patients and families in treatment decision-making. The first can be achieved by including CDG in medical board examinations, disseminating information about CDG through public talks and publications (2.1a), and adding CDG to diagnostic checklists:

*“We should get it [CDG] on a checklist and if they see a child with, for example, an unexplained developmental delay, then they send with all the analyses being requested also a request for a CDG analysis.”* (Multiple roles 11).

Developing quality care approaches to CDG requires identifying successful care practices, testing them among specific CDG groups, discussing the results with colleagues internationally and disseminating selected best practices (2.2a–b).

Finally, to enable patients and families to get involved in treatment, it is necessary to empower parents to clarify doubts (2.3a), and provide them with intelligible information and the opportunity to make decisions:

*“I think that parents also need to educate the physicians. In the hospital where I’m working, I’m also continuously fighting against my colleagues for not explaining things to parents. (…) But I think it’s really important for all parents here to say: ‘OK, I don’t understand. Please explain it to me again, again and again’.”* (Multiple roles 12).

### Digital innovations

Participants stated the need to create an online platform with “state-of-the-art” knowledge on CDG, and to make that expertise accessible to patients, families, health care professionals and researchers worldwide (3.1a) in order to mitigate the negative effects of information scarcity and bolster research. They also considered the possibility of transforming it into a global patient registry enabling the collection of patients’ clinical and biological data:

*“It would be a great idea to create a website where everybody, internationally, could enter their information and you can have questionnaires and a patient registry.”* (Multiple roles 11).

To maximise the online CDG platform’s effectiveness and reach, it should have an international scope, secure funding, employ staff and be multilingual:

*“Those kinds of (online) platforms, when you don’t have the financial means and time to maintain them, they are hard to keep. If you could do that, in an international setting, that would be better because then you can tackle the language problem.”* (Researcher 2).

Participants also emphasised the importance of engaging stakeholders from different countries (3.1b), and expressed concern regarding the need to control the quality of data (3.1c) and to guarantee its security by safeguarding families’ data ownership:

*“If you can get government support and if industry can be interested, great! (…) I think the important thing is that you [patients/families] own the data. This is your data.”* (Multiple roles 14).

## Discussion

This study set out to assess the needs of people affected by CDG and to elicit innovative strategies to address them using a think tank methodology that engaged stakeholders from twenty countries across the globe. Our findings revealed four types of unmet needs: information, health care, psychosocial and representation needs. The former three attest to the mismatch between patients’ multi-level needs and the services available to them. They also suggest that, albeit laudable, rare diseases policy in European Union Member States [31] and the United States [32] has so far proved insufficient to eliminate inequalities in access to quality care among people affected by CDG. Representation needs evidence the difficulties involved in engaging vulnerable groups in participatory initiatives aimed at promoting their rights and interests [22, 33]. CDG patients and families are represented by patient organisations in national and international alliances which have a say in rare diseases policy and health care governance. However, representation may be strongly influenced by a few resourceful and vocal patients and families. The voices of the disengaged majority are rarely heard.

Limited access to quality care has severe consequences for CDG patients and families. It undermines their access to prompt diagnosis, which can cause distress and lead to deteriorating health. Moreover, it may be contributing to underestimate the incidence of CDG. This has a negative impact on the amount of resources allocated to professional training, service delivery and research, thus reducing the availability of information, specialised care, rehabilitation services and treatment. Although some families are able to seek cross-border care services, this may result in delays in obtaining necessary care and cause additional financial strain (e.g. inability to obtain expenses reimbursement) [34]. CDG families are also confronted with psychosocial burden. Uncertainty about disease prognosis, lack of effective treatments, costly therapies and feelings of isolation take a major toll on families’ quality of life. These findings are supported by studies focusing on people affected by other rare diseases, and their caregivers, who also report unmet needs for information, health and social care [6–9].

Bridging and filling the equity gap in access to care for people with rare diseases requires patient-oriented research that can inform decision-making concerned with care quality improvement [35]. It also demands involvement of multiple stakeholders. Sustainable quality improvement entails a collective effort grounded on networks of people and actions aimed at making care responsive to patients’ needs [12]. Our study has made a first attempt at rallying such a network by bringing together lay and professional experts from around the world to elicit community-framed innovations to improve the quality of care and life of people affected by CDG.

Participants proposed civil society, care-related and digital innovations, which include peer-support communities, web-based information resources and an online expertise platform. Altogether, these innovations can redress system failures by facilitating access to a “virtual empowerment toolkit” [6] that can foster knowledge sharing, improve disease management, increase quality of life and promote a sense of community that is crucial for public and patient involvement [10, 36, 37]. Such an empowering device might also help to promote people-centred adjustments to health and social care provision by drawing awareness and providing the impetus necessary to invest on tailor-made professional training, accessible diagnostic tools, intelligible information, optimal management of multi-disciplinary care delivery and dialogic patient/family-provider relationships [38] enabling shared treatment decision-making.

The social innovations proposed by participants also carry the potential to enhance fundamental and translational research by connecting patients, professionals and researchers, and enabling the donation of patients’ clinical data and biological material for research. Some of these innovations are starting to emerge. Rarecommons.org, for example, is an online platform recently created to collect data and develop research on rare diseases through the participation of families and physicians. Digital innovations such as this one can facilitate the co-production of knowledge and technology with the participation of citizens. However, they are not a panacea and they carry ethical, legal and social implications (ELSI). Both lay and professional stakeholders need to be involved in decision-making about ELSI regulation and management [39–41], particularly in regard to issues related to confidentiality, informed consent, ownership, donation, storage and sharing of biological, clinical and personal data, and the return of research results to the public [42]. In the case of CDG and other groups who have been at the margins of decision-making, this may require the creation of participatory spaces specifically designed to enable all stakeholders to voice their concerns, find middle ground and jointly deliberate on policies, care and research sensitive to their needs, values and preferences [43]. In other words, it may require spaces in which formerly detached groups can come together to establish bidirectional partnerships [44] and uncover the elements of expert and patient knowledge that are key for the co-production of patient-centred care and research.

Participants in our study stated that broader representation is critical to achieving equity in care and reducing potential research biases resulting from skewed population samples. As other studies with marginalised groups suggest, creating supportive participatory environments, attending to participants’ motivations for involvement, facilitating access to resources and providing opportunities to effect change are key to enabling inclusive public and patient involvement [2, 22, 33]. However, in the specific case of CDG patients, this may not suffice to achieve broad representation. CDG is an early onset chronic disease that can cause considerable impairment, not least to speech. This makes it challenging to involve large groups of patients using conventional research methods. More creative methodological designs are needed to engage CDG patients in decision-making and research. One option might be the use of creative visual methods (e.g. photography, video, acting performance) [45]. Far from being a “magic bullet” [46], these methods can nonetheless help the CDG community to participate more fully in knowledge co-production and care quality improvement.

### Strengths and limitations

The use of a think tank methodology is a key strength of this study. Meeting face-to-face allowed participants to listen to and reflect about each other’s contributions enriching the depth of the data collected. Furthermore, it afforded CDG stakeholders from various backgrounds and nationalities an unprecedented opportunity to share their experiences and confront different points of view. A post-conference satisfaction survey including 33 out of the 48 think tank participants showed high receptivity to this initiative, with 80% of participants recommending a follow-up in the upcoming world conference.

This study also has some limitations. First, it does not include representatives of disengaged CDG patients and families. Previous studies have found that people with low education tend to report fewer unmet care needs than higher educated people [9]. Most participants in this study were highly educated, suggesting it may be providing a comprehensive depiction of the needs experienced by the CDG community. Nevertheless, to overcome this limitation and frame a fair set of priorities for action, it is essential to carry further research engaging the diversity of patients and families affected by CDG. A second limitation of the study is associated with think tanks being conducted in the English language. This may have favoured native speakers in setting forth their views even though all participants were able to express themselves.

## Conclusion

This is one of the few studies to engage lay and professional experts globally to collectively assess the needs of people affected by CDG and devise social innovations to address them. The social innovations proposed have ethical, legal and social implications that need to be assessed and regulated with the involvement of all stakeholders. To foster the participation of people affected by CDG in the governance of care and research, it is necessary to create participatory spaces where they can fully express their views. That may require the use of innovative strategies and methods to recruit and involve a diverse section of the CDG community in decision-making concerned with the promotion of patient-centred care.

## Abbreviations

CDG: Congenital disorders of glycosylation
ELSI: Ethical, legal and social implications
